# Implementation of Harmonized Food Consumption Data Collection in the Balkan Region According to the EFSA EU Menu Methodology Standards

**DOI:** 10.3389/fnut.2021.809328

**Published:** 2022-01-20

**Authors:** Mirjana Gurinović, Marina Nikolić, Milica Zeković, Jelena Milešević, Agnes Kadvan, Marija Ranić, Maria Glibetić

**Affiliations:** ^1^Institute for Medical Research, National Institute of Republic of Serbia, Centre of Research Excellence in Nutrition and Metabolism, University of Belgrade, Belgrade, Serbia; ^2^Capacity Development Network in Nutrition in Central and Eastern Europe (CAPNUTRA), Belgrade, Serbia

**Keywords:** dietary exposure assessment, food consumption, EU menu, dietary software, research infrastructure, Balkan region, harmonized methodology, sustainable food systems

## Abstract

Initiatives in the Capacity Development in Nutrition Research in the Balkan region in the last decade have been toward the creation of contemporary, harmonized Research Infrastructure (RI) compliant with European standards. This study describes the process of creation and implementation of the European Food Safety Authority (EFSA) EU Menu methodology in the Balkan region during the EFSA support projects for food consumption data collection in four countries (Serbia, Montenegro, Bosnia and Herzegovina, and North Macedonia). This process entailed the application and improvement of an innovative tool, the DIET ASSESS and PLAN (DAP), a platform for standardized food consumption data collection and dietary intake assessment. DAP comprises computerized food consumption, anthropometric measurements, and physical activity questionnaires, validated food picture book, and FoodEx2 exposure hierarchy with sets of facet descriptors of the interest. It hosts the Balkan food platform with a Serbian food composition database (FCDB) and Regional FCDB, compliant with European Food Information Resource (EuroFIR™) standards. The implementation of the DAP platform in national dietary surveys conducted with the support of the EFSA EU Menu project in Balkan countries enabled harmonized food consumption data compilation and reporting. Application of the methodology entailed the development of study protocol and extensive education and training of study personnel. The entire data collection process was managed by internal and external survey coordinators. A pilot study was conducted to test the entire data collection and control process and was afterward used to make necessary improvements and adjustments to meet EU Menu requirements. Data collected are internationally comparable with food consumption data in other European countries within the framework of the EU Menu program. The existence of such data in the Balkan region will catalyze research activities in emerging topics, such as identification of dietary patterns, the establishment of national nutrient reference values and food-based dietary guidelines (not only in Serbia, but in the whole Balkan region), dietary exposure assessments, the endorsement and evaluation of new food legislations, the environmental and other effects of diet on the food system. The developed and implemented methodology underpins evidence-based policy-making processes lacking in the field of public health nutrition in the region.

## Introduction

Current dietary patterns are unhealthy, unsustainable, and inequitable for many populations, and present a significant risk factor in the global burden of disease and death (1). The analysis of the impact of inadequate consumption of major foods and nutrients across 195 countries on mortality and morbidity rates of non-communicable diseases, identified diets high in sodium and sugar, and low in whole grains, fruits, nuts and seeds, vegetables, and omega-3 fatty acids as the leading risk factors in mortality, each contributing more than 2% to global deaths (2). Non-optimal intake of three dietary factors (whole grains, fruits, and sodium) accounted for more than 50% of deaths, and 66% of disability-adjusted life years (DALYs) were attributed to diet (2). Six of the top 11 risk factors driving the global burden of diseases are related to diet (3). The triple burden of malnutrition—undernutrition, overweight, and obesity, as well as micronutrient deficiencies—are present to varying degrees in all countries of the region in Europe and central Asia (4). Monitoring policy implementation in the WHO European Region shows that there is a lot of improvement in the food and drink environment, but there is still a need to encourage member states for food system transformation (5).

The Yugoslav study of atherosclerosis precursors in school children in Serbia (6, 7), conducted in 1998–2003, was the first and (until now) has remained the only national survey in Serbia based on comprehensive dietary assessment methods that collected data on food consumption, energy, and nutrient intake of families and children (7). To overcome the evident lack of comparable and harmonized food composition and dietary intake data in the Balkan region, the application of internationally accepted, validated indicators, and standardized methods was warranted (8–12). National dietary surveys at an individual level are already carried out in many European countries and provide valuable information for use in national policy decisions, and are important in monitoring dietary habits, in nutrition surveillance and in conducting dietary, contaminant, and chemical exposure assessments. The availability of harmonized and detailed food consumption data at the European level has been widely recognized as essential to improve the consistency and reliability of exposure assessments carried out by the European Food Safety Authority (EFSA) Panels at the European level. Therefore, the EFSA has created guidance on methodological principles to follow and protocols to be used for individual dietary surveys within a pan-European context to obtain a more harmonized food consumption database (13).

Adequate Research Infrastructure (RI) in the food, nutrition and health domain, and Capacity Development (CD) is essential for nutrition epidemiology, innovative nutritional research, and effective public health nutrition strategies to address the diet-related diseases and malnutrition (8, 14). Harmonized food consumption data are a foundation for different research topics important for the improvement of food system elements, which are all interconnected, as presented in the Food System Dashboard (15). The development and adoption of novel research and innovation (R&I) approaches will provide evidence to inform food system transformation and will serve as catalysts for change (16). RI allows the accurate evaluation of dietary patterns, nutrient intake, exposure assessment, etc., and ensures data comparability at the international level. The existence of harmonized food consumption data is a prerequisite for research in the field of the diet-health relationship, the design and implementation of various agro-food policies and regulations (17, 18), and a basic resource for decision-making in the field of food system transformation.

The lack of capacity and the inconsistent situation in the area of food consumption surveys in Central and Eastern Europe/Balkan countries (CEE/BC) was recognized by the United Nations University (UNU), Food and Nutrition Program, and the UN Standing Committee on Nutrition (SCN), who encouraged the formation of NCDNCEE (Network for Capacity Development in Nutrition in Central and Eastern Europe) in 2005, which became CAPNUTRA network in 2012 (19).

Capacity Development in Nutrition Research in the Balkan, in the last decade, initiated the creation of contemporary, harmonized RI compliant with European standards (19). One of the fundamentals of this RI is an innovative tool, DIET ASSESS and PLAN (DAP), a platform for standardized and harmonized food consumption collection, comprehensive dietary intake assessment, and diet planning (20). The DAP platform and its building blocks came as a response to the identified lack of food composition datasets, food consumption data, dietary recommendations, and the general need for investments in capacity building in nutrition research.

Numerous network's activities and research on the existence of various food system elements in CEE/BC were focused on the harmonization of tools needed for high-quality food consumption surveys, such as dietary software, the food composition database (FCDB), questionnaires, survey protocol, and food picture book.

The CAPNUTRA network has been working on the creation of contemporary, harmonized RI compliant with European standards. An innovative tool, the DAP, a platform for standardized food consumption data collection, and dietary intake assessment, as an important RI element, has been continuously updated to satisfy the needs of harmonized food consumption data collection in the Balkan region (20). It comprises computerized food consumption questionnaires, a food picture book, and the FoodEx2 catalog. In addition, the DAP hosts the Serbian FCDB and Balkan food platform with Regional FCDB, compliant with EuroFIR™ standards (17, 21, 22).

These activities have been timely overlapped with the EFSA call for EU Menu surveys in instrument for pre-accession assistance (IPA) countries, which was recognized by the network as a chance for the fulfillment of its plans for harmonization. In 2017, four Balkan countries from the CAPNUTRA network (Bosnia and Herzegovina, Montenegro, North Macedonia, and Serbia) were successful in applying for EFSA Support (23). Adaptation of the platform was performed to enable the implementation of the methodology according to the EU Menu and to allow international comparability. Besides the application of the DAP, the methodology entailed development of study protocol and extensive education and training of study personnel.

Harmonized food consumption data collection in the Balkan region will catalyze research activities, aligned with EFSA requirements, in emerging topics, such as identification of food consumption patterns, the establishment of national nutrient reference values and food-based dietary guidelines (not only in Serbia, but in the whole Balkan region), dietary exposure assessments, evaluation of new food legislations, the environmental impact of diet, and other topics within the food system. Finally, developed and implemented methodology, together with the DAP platform, underpins evidence-based policy-making processes lacking in the field of public health nutrition in the region.

This article describes the steps of harmonized implementation of the EU Menu guidance in Balkan region and to present the adjustments made to allow simultaneous use of the same tools in the food consumption surveys in Bosnia and Herzegovina, Montenegro, North Macedonia, and Serbia.

## Materials and Equipment

European Food Safety Authority (EFSA) support for EU Menu projects, awarded to four countries from the CAPNUTRA network, anticipated harmonized data collection beyond the EU Menu guidance, since all of them have been proposing the use of the DAP platform (23). Moreover, the CAPNUTRA network focused on the adaptation and implementation of a research methodology according to EU Menu guidance by developing the tools that will be used by all four countries (questionnaires, protocols, training, centralized support to interviewers, etc.) in a standardized and harmonized way. The DAP was implemented in Balkan region within the EFSA projects—Support to National Dietary Surveys in Compliance with the EU Menu methodology (sixth and seventh support)—“The adults' survey,” including subjects from 10 to 74 years old, 2017–2021 (Serbia, Bosnia and Herzegovina, and Montenegro) and “The children's survey,” including subjects from 3 months up to 9 years old, 2017–2021 (Serbia and North Macedonia) and Montenegro (seventh support) 2018–2023.

### Development of the Survey Package

The survey package contains a written consent form, a general questionnaire, double 24-h-recall/food record forms, Food Propensity Questionnaire (FPQ), and International Physical Activity Questionnaire (IPAQ) (24). It is created to fully cover all variables, together with their predefined list of answers (following the EFSA nomenclature) stated as mandatory in the EFSA data transmission schema (13). The general questionnaire aims at collecting basic information, such as date, weekday and season of the interview, the gender and age of the subject, as well as sociodemographic characteristics, such as region, residential place, settlement type (rural/urban), religion, ethnicity, household size and composition, marital status, employment status, occupation, and education of the subject or of the parent/caretaker (in the case of children). In addition, this questionnaire contains a list of information related to the health status of the subject, such as allergies, chronic illnesses and therapies, pregnancy/lactation, type of usual diet, and smoking status. Finally, at the end of the general questionnaire, there is a list of questions related to the anthropometric measurements, such as weight, height, waist and hip circumferences, and blood pressure.

### 24-h-Recall and Food Record

A 24-h recall and food record were used as principal food consumption data collection instruments for the adult and children population, respectively, as proposed by EU Menu guidance. To facilitate accurate and complete recall and overcome respondents' memory limitations, the formal structure of the 24-h recall and food record followed a stepwise procedure. The multiple-pass questioning method, tailored in accordance with cognitive principles and practical research experience, encompasses sequential interview iterations based on a predefined pattern. Although particular protocols of this staged approach may differ across studies in a number of phases and their scope, the basic methodological concept remains the same. The application of combined structured and unstructured interview steps, coupled with numerous memory cues, is designed to encourage individuals to provide a precise, truthful, and detailed dietary report (18, 25, 26). Within the EU Menu study, the 24-h-recall stepwise procedure comprised five passes. In the first (unstructured) step, the respondent was asked to provide an uninterrupted recall of the food consumption during the previous day, not essentially in chronological order. After this phase, subjects were questioned for any forgotten or omitted food items. The third and fourth steps denoted probing questions targeting details of the intake (including specifics regarding the time and occasion of consumption and quantities consumed). In the final step, skilled interviewers carefully reviewed the list of reported food items with the respondent and, if appropriate, complemented the previously created report with additional foods, eating occasions, or other relevant details.

The general part of the recall/record was developed to collect information on the date of the consumption, season, weekday (working or weekend day—predefined in the survey calendar), specifics about the daily consumption (more than usual or less than usual), number and type of eating occasions/meal.

Regarding food items reported in the interview, the form requires the following information: type of meal (breakfast, lunch, dinner, etc.), time and place of food consumption occasion, food name, whether it is a recipe or not, and consumed amount. To collect complete information about all consumed food/beverage items, the form was extended with questions related to the preparation process, qualitative information (fat and/or sugar/salt-related info, etc.), presence of any sweetening or fortification agents, and packaging material. In this way, the collection of information needed for the attribution of mandatory facets for FoodEx2 classification, proposed by EU Menu guidance, was ensured. The consumed portion sizes were estimated based on natural units, household measures, packaging information, and country-specific portion size measurement aid, i.e., the previously tested Food Atlas described below (22).

On the field, interviewers collect information on required FoodEx2 food facets if applicable for every food item, and document it on the paper format. Later on, this information is inserted on the platform in the provided interface (**Figure 3**).

To assure the adequate quality of the obtained data, at the end of the recording period, within debriefing sessions, the form contains an additional set of probing questions that interviewers use to review the recall/record with the subject or parent/caretaker and clarify possible omissions and uncertainties, probe for Supplementary Material, or make necessary modifications.

### Food Propensity Questionnaire

The project-specific FPQ forms have been developed to collect information on the frequency of food consumption for adults and children in the Balkan region. They were designed to measure habitual consumption over the past year for adults or the past 3 months for children. They are intervieweradministered during the face-to-face interview with the subject or parent/caretaker. The informative, culturally and age-appropriate food list was assembled by the experts in the field of nutrition research. It was constructed based on previously conducted surveys (27, 28), expert consultation, and similar questionnaires developed by countries that have already completed EU Menu surveys.

The instrument comprised 66 and 64 food items clustered in 15 and 14 food groups for adults and children, respectively. An additional part of the FPQ referred to the intake of dietary supplements and was created in accordance with the form provided by EFSA. Being a qualitative instrument, the FPQ was primarily used as a quality control tool for checking the answers from the 24-h-recall/food record and as a supportive research instrument designed to capture sporadically and seasonally consumed food items. The form of the FPQ is simplified, as suggested by EU Menu methodology guidance to reduce the burden on participants.

### International Physical Activity Questionnaires

The purpose of the IPAQ (24) is to provide a set of well-developed instruments that can be used internationally to obtain comparable estimates of physical activity. There are two versions of the questionnaire: The short version is suitable for use in national and regional surveillance systems. In the survey pack, the short version was applied, providing basic information on frequency and length of intensive, moderate, and walking activities and sedentary time on an average weekly basis.

Prior to application, the IPAQ was translated to national languages with an aim to assure conceptual consistency of the instrument across all the participating countries, and the responsible research organizations employed the same, harmonized translation procedure (29). The first step was the forward translation performed by two independent certified translators. The encountered semantic discrepancies, idiomatic issues, and other incongruities between their versions were discussed and resolved among an expert panel comprising a bilingual reviewer, a health-related research representative, and a person with expertise and experience in the field of questionnaire development. Upon reaching the consensus, backward translation to the original language was performed by independent translators to ensure the accuracy of the previously approved version. The prefinal form of the instrument was pilot tested on a small sample of intended respondents.

### Harmonization of Data Collection for the Anthropometric and Physiological Indices

The harmonization of data collection for the anthropometric indices and blood pressure between countries was addressed by the creation of a detailed manual for field workers with both textual instructions and supportive graphical presentations. Furthermore, although national organizations were accountable for the selection and allocation of measurement equipment, i.e., scales, tensiometers, and stadiometers, their precision was predefined and consistent in all countries.

### Food Picture Book Developed for the Balkan Region

The food picture book is one of the crucial instruments in food consumption surveys. Due to diverse dietary habits, it is usually developed on the national level. However, given that dietary habits in Balkan region do not vary a lot and that the food market is very common, CAPNUTRA has decided to develop a unique food picture book to be used in all four countries and in this way add up to the harmonization of the data collection between the countries.

In 2018, the “Food Atlas for portion size estimation in the Balkan countries” was developed and validated for application in dietary surveys conducted in Balkan region (22). Based on previously conducted food consumption surveys, the most commonly consumed simple foods and recipes were selected for the food atlas (7, 27, 28, 30). To address local dietary patterns and improve the cultural competence of the atlas, additional dishes from traditional cookbooks and restaurant menus from all four countries were included. The final list of 125 food items and utensils was reviewed and approved by the panel of experts in nutrition and dietary intake assessment. The Food Atlas was published in limited circulation, for study purposes, and was distributed in the form of a PDF to some participants (parents or participants with whom a second interview was arranged *via* phone). This publication is now available open access food and nutritional tool (in Serbian language) on CAPNUTRA website: https://www.capnutra.org/food-and-nutritional-tools/.

### Food Composition Database

CAPNUTRA has been working for a long time on the creation of the Balkan food platform, including the Regional FCDB, according to the EuroFIR™ standards (8) and on building capacities for FCDB development in the region (8, 20, 31).

Prior to EFSA EU Menu projects, the Regional FCDB for Balkan countries passed through significant improvements in the context of the functionality of the food composition data management system. The system is adjusted for regional languages, as well as for English, the procedure of nutrient data documentation is harmonized, and recipe calculation with yield and retention factors is included. The FoodEx2 exposure hierarchy with facet descriptors is incorporated and implemented in the food description process, which made this FCDB completely adapted for the defining of new food items and for food recall data collection in the format required by the EU Menu project.

During the EFSA EU Menu projects in Balkan region, national research teams were responsible for inserting new food items, recipes, and supplements into the FCDB, which were identified and reported by the interviewers during the study. To assure a high level of control, experts in nutrition from the Institute for Medical Research, Center of Research Excellence in Nutrition and Metabolism (IMR-CENM), and CAPNUTRA teams had a centralized role in checking the quality of inserted data. All the changes and updates in the FCDB are automatically visible in the DAP software and are accessible for all Regional FCDB users.

### Update of the DAP Platform According to the EU Menu Guidance

The DAP platform represents an important RI for nutrition epidemiology and public health nutrition research and consists of the tool developed for diet planning and consumption questionnaire evidence (24 h, FFQ, and FPQ questionnaires), the Food Composition Database Management (FCDM) tool, containing the Serbian and Balkan Regional FCDB and Nutrient recommendation datasets (20). It presents one of the new technology-based tools for dietary intake assessment (32).

Initially, the DAP software was evaluated in the EFSA Ring Trial, which took place within the EFSA project: “Dietary monitoring tools for risk assessment” in 2014 (29, 33). The aim of the project was to identify and evaluate available data collection protocols and tools for capturing food consumption information for risk assessment purposes in Europe (32). The DAP was developed according to EuroFIR™ guidelines (34) and performs comprehensive calculations, combining data from external datasets: food composition database(s) and nutrient recommendation datasets. It enables extensive evaluation of dietary intake on individual and/or population levels, menus and recipes, food group consumption, diet planning, food design/reformulations, food labeling, etc. (20).

Upon receiving the support from EFSA to conduct national food consumption surveys in four WBCs in 2017, the DAP software significantly evolved toward harmonization with EFSA guidance on the EU Menu methodology.

The electronic versions of standard food consumption questionnaires developed according to the EU Menu methodology as described above were implemented in the software: 24-h dietary recall/food record and FPQ. The 24 h, FFQ/FPQ interfaces for data input with the related reports on the DAP platform were made available in different languages: Serbian or other regional languages (Macedonian, Bosnian, and Albanian) and English (default), and they can be easily adapted and used in any other language.

The DAP software structure is updated to allow the input of food, beverages, and food supplements consumed during the survey days in accordance with common 24-h food recall/record rules and requirements set by the EU Menu methodology. During the data entry, it is possible to automatically search, describe, and quantify each item using adequate quantification methods, followed by probing questions and forgotten item questions. Furthermore, the brand of food, time, place, and company/other activities of each consumption event can be recorded. The DAP is propped up with the food atlas, containing country-specific portion sizes and household measures, corresponding to the foods in the Regional FCDB. The photo set from the food atlas is integrated into the software. The food atlas is available for interviewers in the paper format as well.

Apart from the LanguaL™ (35) as a standardized coding system, which has been integrated into the DAP since its first version, the DAP structure is upgraded with the FoodEx2 (36) exposure hierarchy, which enables the matching process on the level of foods and recipes, and, subsequently, on dietary questionnaires (facet descriptors). This makes the DAP program a concurrent tool for large nutrition epidemiology studies.

The DAP software allows recipe collection and application of yield and retention factors by applying appropriate cooking methods, predefined in the systematic calculation. Implementing the yield factors, the software calculates the weight changes of the recipe ingredients (weight gain or loss), which may occur during the applied cooking method. With the retention factors, the software calculates the changes in vitamin and mineral contents of the recipe ingredients, depending on the implemented cooking treatment. All self-made composite dishes could be disaggregated and described on the component/ingredient level based on information provided by the subject.

The DAP software features for the collection of data on dietary supplements' consumption have been improved, as well. The platform now allows the collection of detailed information on the respondents' use of food supplements. This information can be collected at the level of both dietary intake assessment instruments, i.e., FPQ and 24-h recall/record. In the FPQ, a list of the most frequently used types of food supplements is presented in an interactive table with nine offered consumption frequency intervals, along with options for specific information on dosage, brand name, product type, and packaging of the supplement in question. Initially, the examinees were asked about the use of supplements from the predefined list. If necessary, new supplements mentioned by the examinee were added into the table along with accompanying data. In 24-h recall/food record, individual supplement consumption during the days for which data are collected was reported, including information about the supplement form to allow its complete description in FoodEx2.

**Figure 1:**
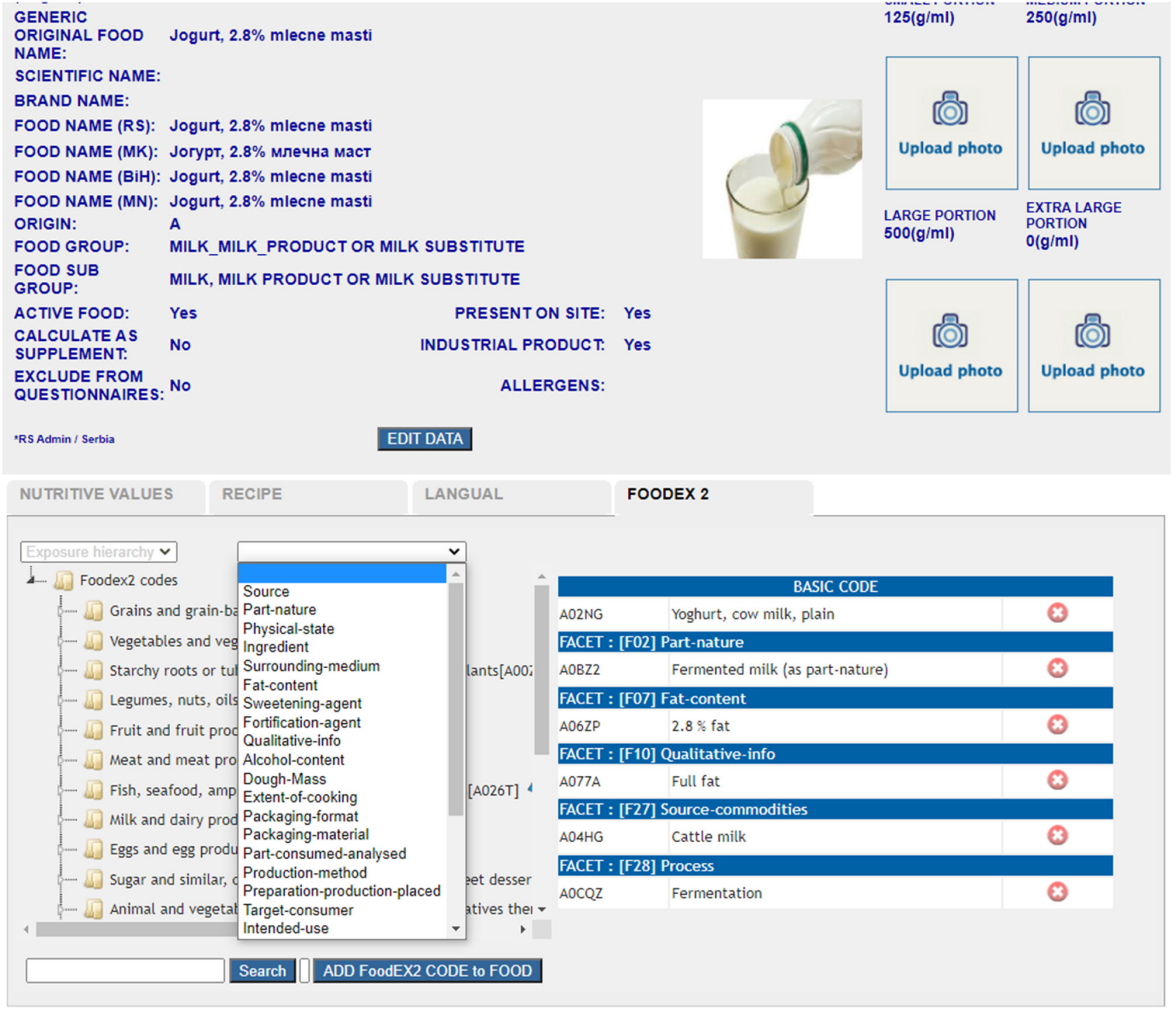
FoodEx2 coding on the level of the food item incl. all required facet descriptors.

**Figure 2:**
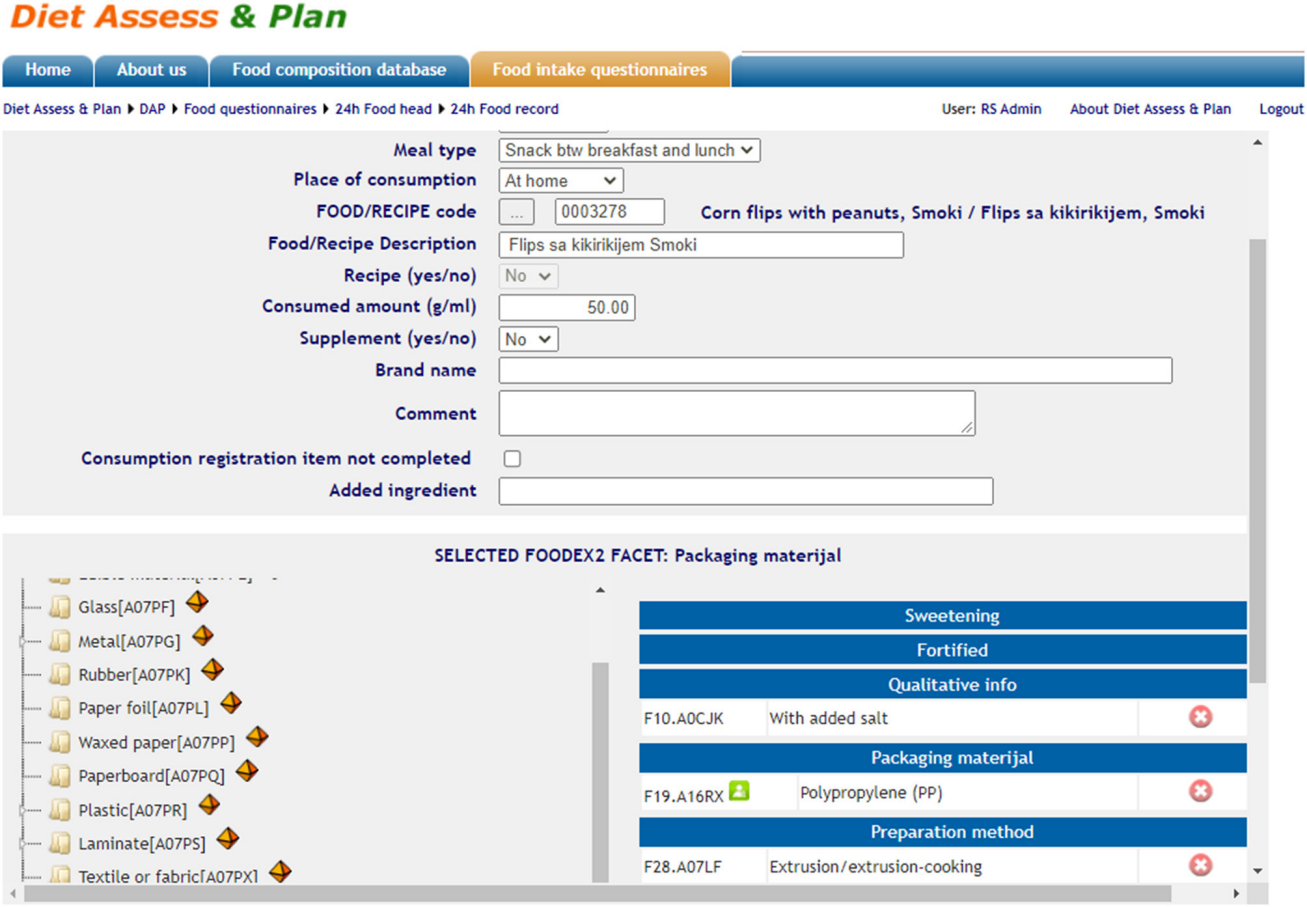
FoodEx2 facet descriptors addition on the level of individual consumption.

Furthermore, the DAP has been improved with several options implemented for automatic quality checks during the data input—mandatory fields marked if not filled in and outlier value indicators for energy and macronutrient intake by subject per day. Based on the requirements of the EU Menu survey, the DAP platform was customized to cover the interview protocol and to support the CAPI method (Computer Aided Personal Interviewing). The protocol requires the registration of general data for each examinee (demographic, consumption habits, anthropometry, and data related to food allergies). All parts of the food consumption questionnaire were harmonized with the thesauri system presented within the EU Menu data transmission schema. The data exported from the software cover the EU menu data coding standards: for the general data (labor, education, profession, gender, special condition, special diet, etc.), consumption data (weekday, season, exception day, meal-type, place, etc.), and for food items (amount, ingredients' raw and cooked amount, FoodEx2 code for the recipe, FoodEx2 code for raw foods, nutrient content information, etc.). The search engine based on FoodEx2 tree structure was available for compilers (Figure 1) during the creation of new food items in the database and for interviewers in 24-h food record questionnaires during the registration of each consumed food item reported by the examinee (Figure 2).

### Protocol Development

To facilitate straightforward usage of developed tools and the application of EU Menu methodology, a unique Survey Protocol has been created for interviewers and their supervisors. It gives precise instructions on interactions with participants, the methodology of conduction of each questionnaire and review of collected data, anthropometric measurements, specific requirements for fieldwork with the child population, a manual for the usage of the DAP platform (data entry, quality control checking process, etc.), the usage and update of FCDB, FoodEx2 coding within the DAP platform, etc. The survey protocol was translated to Macedonian and Albanian for its application in North Macedonia and used as the training material before launching the pilot studies. The manual for the usage of the DAP platform is also available online for the users (with the access credentials).

## Methods

### Capacity Building

CAPNUTRA played a leading role as an advisory for professional capacity support since the beginning of the application process for the EFSA EU Menu project. Partner countries used CAPNUTRA support and guidance to prepare their project proposals and plan further realization based on the devised infrastructure and methodology offered by CAPNUTRA. The development of RI was followed by overall capacity building for food consumption data collection. The CAPNUTRA team organized several workshops in which national partners were introduced to the aims and methodology of the study, which is to ensure a harmonized way of data collection.

A series of workshops and training took place in 2017, prior to the pilot study initiation: In Belgrade [CAPNUTRA Symposium-Capacity development in dietary intake survey harmonization with EU Menu methodology, regional training for DIET ASSESS and PLAN (DAP) application and implementation in West Balkan countries (WBC), 11–12th September 2017 Belgrade, Serbia] (37), following trainings in Mostar (B&H) and Podgorica (MNE). The aim of the trainings was to introduce partners and potential users of the DAP platform to the entire system, survey pack, and protocol of the study. During these training sessions, potential interviewers, data administrators, and coordinators were provided with comprehensive knowledge and hands-on practice on how to conduct the study, use the DAP platform, and ensure the quality and completeness of data.

The trainings were comprised of:

a. Introduction to survey package—questionnaires and study instruments, and how to appropriately use them to collect data from participants on the field.
b. Simulation of face-to-face and telephone interviews, with a focus on how to correctly collect all relevant information, such as anthropometric measurements, physical activity, FPQ, multi-pass approach in 24-h recall.
c. Introduction to work with the FoodEx2 system, catalog browser, and EFSA Interpret and check tools (for data administrators).
d. Application of the DAP platform for interviewers (questionnaires input) and data administrators (data quality check, new food items insert).
e. Hands-on food atlas application in real-time and during the data insert.
f. Knowledge assertion and certification.

The training was organized as a personalized course, i.e., a face-to-face individual continuous training system. Interviewer training consisted of protocol presentation, role-playing (conducting practice interviews), panel meetings, and individual sessions (on-site and on-line) with study core team members.

The training covered the aim of the survey, description of the methodology, overview of the questionnaires, features and functions of the DAP platform, FoodEx2 coding system and recipe management, challenges to be expected and practical solutions during the conducting of the interviews, anthropometry measurements, data management, and practical exercises.

Written didactic materials, research protocol, and the possibility of consultation with competent supervisors were available to interviewers throughout the survey. Several additional online training sessions were provided for group or individual interviews or data administrators. Special training and webinars were organized for data administrators to prepare them for FoodEx2 coding, the data sorting process, and report files preparations, both in the Pilot and in the main study.

CAPNUTRA provided continuous guidance for all national study teams in all of the phases of the study, including on-call IT support and updates.

CAPNUTRA also organized a symposium on sustainable food systems for healthy diets in countries of Central and South Eastern Europe (CSEE) with integrated training in food consumption data collection and strengthening the thematic regional networking on October 15–17, 2018 in Belgrade, Serbia (37), where the EU Menu methodology and its application were further presented to an international research audience.

### Recruitment and Selection of the Fieldwork Staff

To ensure the quality of the study, it was important to recruit personnel with an adequate knowledge background. During the preparatory phase for the pilot study, the CAPNUTRA and IMR-CENM team used professional contacts to recruit interviewers who had MSc or PhDs in the field of nutrition, or other professionals with an extensive nutrition-related background. The selected persons were invited for the initial meeting, where the IMR-CENM team introduced them with the aim of the survey and presented the survey pack, study protocol, and the survey calendar. To meet the study needs, additional recruitment and training sessions were organized throughout the entire study. Similar selection criteria were applied in other WBCs, where the study team consisted of personnel with food- and nutrition- related backgrounds and a comprehensive understanding of the study requirements.

### Pilot Study and the DAP Platform Adaptation

The pilot study was an essential step in the study, as it provided an opportunity to test the functionalities of the DAP platform in real-time and to adjust the methodology and workflow, personnel efficacy, the data collection process, and all relevant aspects of the study.

Since the pilot study commenced in almost the same period in 2017 in all Balkan region using the DAP platform, the IMR-CENM team used this opportunity to simultaneously adjust different aspects of the methodology. Different errors on the platform were identified during use, and they were corrected simultaneously. Several foods and utensils were identified as missing in the Food Atlas, which resulted in the upgrade of the final version of the Food Atlas.

In these processes, the IMR-CENM team collaborated closely with other national coordinators and used their feedback to introduce all the necessary adjustments.

### Hierarchy of Quality Control

The IMR-CENM team established a hierarchy of data administrators to ensure that all the required data are collected in the correct and complete format. Thus, there is a central data administrator with permission to access and update any data related to consumption or food items. This administrator can create reports and oversee all the changes in the data on the entire platform.

National data administrators have access to national datasets and national food composition but cannot change any data from other national datasets or food data. They can insert new foods/recipes/supplements and FoodEx2 codes.

Regional administrators have access to all the questionnaires within the assigned region and can make changes and corrections of errors.

Compilers have access to questionnaires assigned to them according to the study calendar, can insert food intake and other collected data, and can withdraw data from FCDB.

## Results

This study presents the implementation of harmonized methodology according to EU Menu requirements for comprehensive food consumption data collection in the Balkan region. It is a massive and fundamental resource of research infrastructure, particularly as there was a lack of food consumption data in the entire region for decades. The study, the implemented methodology, and harmonized data present a basis for further food and nutrition research, policy and strategy initiatives, exposure and environmental impact of food consumption assessment, development of dietary recommendations, and are a high-quality foundation for the establishment of food system research. For instance, the dataset has already been employed for exposure risk assessments to aflatoxin M1 in milk products and nitrites and phosphorus intake from meat products in the child population (38, 39). All four countries successfully submitted food consumption data to EFSA (two national study reports are already available online at the moment) (40, 41), which can now become part of the pan-European pool of harmonized data resources within the EU Menu umbrella (42).

### Validation of Harmonization

To evaluate the quality of the harmonization of the whole system, energy intake for participants in the pilot studies was compared among the countries per age group and gender (Table 1). No statistically significant differences were found, which confirms harmonization of data collection and data processing functions within DAP features.

**Table 1:** Evaluation of quality of harmonization of the DAP system functionalities.

	**Adolescents**						**Adults**					
	**Female**			**Male**			**Female**			**Male**		
Country^*^	***N***	**Mean (kcal/day)**	**SD**	***N***	**Mean (kcal/day)**	**SD**	***N***	**Mean (kcal/day)**	**SD**	***N***	**Mean (kcal/day)**	**SD**
Bosnia and Herzegovina	15	1,950	473	15	2,111	365	31	1,958	472.3	30	2,554	581
Montenegro	16	1,901	569	15	2,196	707	30	1,694	505.5	30	2,289	680.0
Serbia	15	1,758	394	16	2,375	774	29	1,883	507.0	33	2,678	796.0

* *North Macedonia data not shown, as they reported their data independently*.

### Reporting

The DAP platform allows for the processing of data in various forms of reports (20). All the gathered data can be exported in CSV or XLS formats on an individual or group level for every consumption event/season/region, etc.

These reports could be:

➢ Reports on dietary patterns: food consumption/food groups (FoodEx2)/meals % of energy value (EV);
➢ Reports on nutrient intake (energy, macro- and micronutrients intake) and contribution of food group sources to total intake, against DRVs;
➢ Reports on the distribution of energy and protein sources between animal and plant-based food;
➢ Reports on FoodEx2 codes and facet descriptors;
➢ Reports of food intake per person/meal/100 g, total and/or average amount per day;
➢ Reports on respondents' consumption of dietary supplements with specifics regarding the type, pharmaceutical dosage form, nutrient content, time frame, and regimen of intake. Contribution to nutrient intake may be presented both separately and together with regular dietary sources;
➢ Reports on subjects' demographic and anthropometric characteristics, physical activity, and other tailored reports.

Clusters of these reports enable nutritional status and adequacy assessments. These output files can be presented on screen and/or printed automatically, and are created to be functional for statistical analyses. The software allows storage, output, and export of different databases in a standardized way in accordance with the EU Menu data transmission schema.

**Figure 3:**
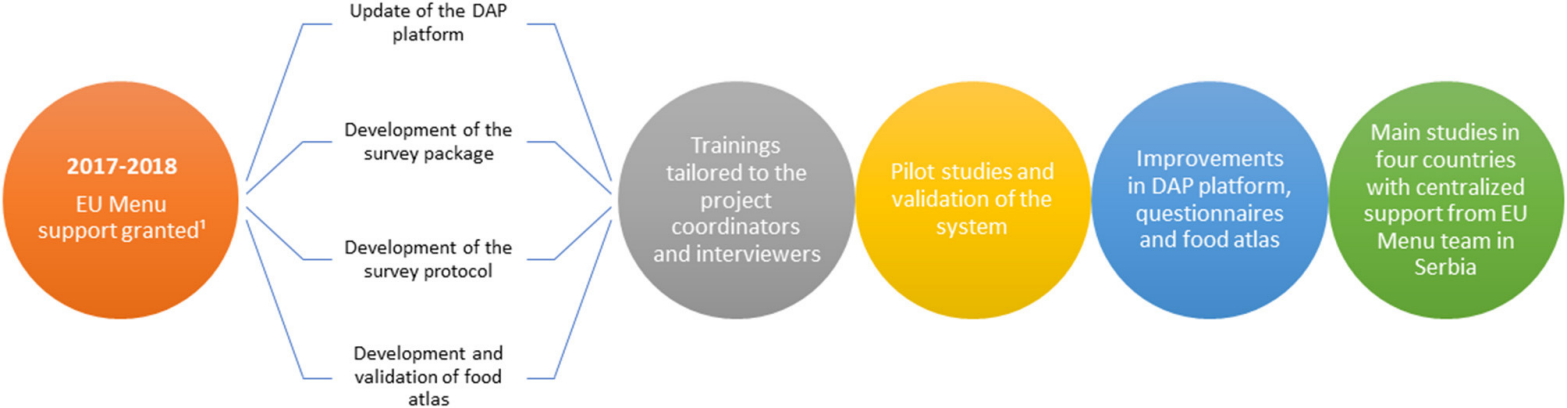
Schematic overview of step-wise approach to implementation of the harmonized food consumption survey in Balkan countries. 1“The adults' survey,” including subjects from 10 to 74 years old (supported in Serbia, Bosnia and Herzegovina and Montenegro); “The children's survey,” including subjects from three months up to 9 years old, 2017–2021 (supported in Serbia, North Macedonia, and Montenegro).

The overall step-wise approach to the implementation of the harmonized food consumption surveys in the region is shown in Figure 3.

## Discussion

Harmonized and standardized primary individual-level dietary data collection, processing, and analysis are imperative for informed policymaking. There are many challenges and gaps on national and worldwide levels on dietary surveillance data (43). The global assessment of food consumption and diet quality poses many challenges. To date, there is no single, validated composite index to measure the multiple dimensions of diet quality across all countries (44). Food insecurity can affect diet quality in different ways, potentially leading to undernutrition, including micronutrient deficiencies, as well as overweight and obesity. Healthy diets are a prerequisite to achieving many SDGs and global nutrition targets. However, global monitoring of diet quality poses multiple challenges. While there are evidence-based guiding principles for healthy diets, it has been difficult to develop valid food- or diet-related metrics of diet quality for global monitoring because of the wide variety of foods consumed and dietary patterns observed worldwide. The scarcity of data on what people eat—especially data that are comparable across countries—adds to the challenge of monitoring trends in diet quality worldwide (44).

In the process of harmonizing food consumption data collection methodology and building a common European food consumption database, the EFSA within the EU Menu project is currently supporting 36 dietary surveys on children and/or adults from 18 European Union Member States and 4 pre-accession countries (23).

The processes of food consumption data collection on the global level are also supported by the Food and Agriculture Organization (FAO) and WHO, who have developed the FAO/WHO Global Individual Food consumption data Tool (FAO/WHO GIFT) (45), an open-access platform that collates, harmonizes, and disseminates existing dietary data from different countries at a global level, with 22 datasets available.

National dietary surveys (NDS) provision across Europe is inconsistent. A recent review has found that less than two-thirds of countries in the WHO Europe region have nationally representative NDS, and most gaps were identified in Central and Eastern European countries (CEEC). Dietary methodological differences may limit the scope for inter-country comparisons (46).

Internationally recognized indicators and harmonized methods compliant with European standards are essential elements for nutritional monitoring and evaluation at national and regional levels, and many international frameworks and action plans included them in the recommendations. The Second International Conference on Nutrition: Framework for Action (ICN 2 FfA) in the recommendation No. 58, urges countries to improve and harmonize the monitoring and evaluation of nutrition data (47). The collection of dietary intake data is essential for evidence-based policymaking in the areas of leveraging the potential of nutrition-sensitive agriculture and food systems for healthy diets and closely tied to Decade of Action in Nutrition and SMART commitment (48). The WHO European Food and Nutrition Action Plan 2015–2020 Objective 4 supports surveillance, monitoring, evaluation, and research, which is to ensure that data from surveillance are accompanied with accurate analyses, interpretation, and evidence-based policy recommendations (49). One of the six priorities in WHO ambition and Action in Nutrition for 2016–2025 is to support the establishment of targets and monitoring systems for nutrition to monitor and evaluate this implementation by defining standards and to develop tools for collecting and analyzing data (50).

Furthermore, key messages from the FAO/WHO Regional Symposium on Sustainable Food Systems for Healthy Diets in Europe and Central Asia, Budapest, Hungary, in 2017 (http://www.fao.org/europe/events/detail-events/en/c/1034293/), convey that “*Governments should take action to ensure that adequate capacities of national statistical services are developed for the monitoring of SDG indicators and to strengthen data collection and analysis for evidence-based policy, including food consumption and nutrition data, surveillance of child growth and nutritional status of the population, food composition data of commonly available local foods, data on food contaminants; develop and support food, nutrition, and health research infrastructure capable of creating an up-to-date, trustworthy base of evidence for policies”* (51).

A recent evaluation of the food system elements status in CSEE countries has identified that seven countries from this region (LT, EE, HU, BG, SI, RS, and CZ) reported that they have established some kind of information system related to food and nutrition that provides data on food systems and nutrition policymaking. In most cases, the information is derived from the food consumption surveys, household budget surveys, and food production and expenditure (52).

One of the specific action points to improve policies and capacity building in food and nutrition stated in the Belgrade declaration for strengthening regional capacities on sustainable food systems for healthy diets and nutrition in Central and South-Eastern European region (Adopted during the Symposium on sustainable food systems for healthy diets in countries of Central and South Eastern Europe (CSEE) with integrated training on food consumption data collection and strengthening the thematic regional networking, on 15–17 October 2018, in Belgrade, Serbia) is that*: “There is a continuous need to further develop and implement standardized monitoring systems, nutrition, and health surveys, aligned with ongoing initiatives, and to support initiatives for establishing a surveillance system for nutrition data collection for evidence-based policymaking”* (52).

Food systems need to be repositioned from just supplying food to sustainably providing high-quality diets for all, which are key to improving nutrition and preventing malnutrition in all its forms. Sustainable food systems emphasize the role of diets as a core link between food systems and their health and nutrition outcomes (53). The transformation of food systems and shifting to healthy and sustainable diets require longitudinal monitoring using accurate dietary intake assessment at individual and population levels, the establishment of dietary surveys, and nutrition surveillance systems with harmonized methodology and validated nutritional tools (54). Making the shift to sustainable nutrition with food systems is one of the cornerstones of FOOD2030 as it is presented in the European Commission (EC) the *Food 2030 pathways for action: Research and innovation policy as a driver for sustainable, healthy, and inclusive food systems* (55). Considering that the region is vulnerable when it comes to providing food safety and security and is less tolerant to shocks and stresses, such as the current COVID-19 pandemic (56), the monitoring of elements of food systems and availability of food consumption data to perform it is of utmost importance.

The FAO and WHO prepared the guiding principles for sustainable healthy diets, taking a holistic approach to diets; they consider international nutrition recommendations, the environmental cost of food production and consumption, and the adaptability to local social, cultural, and economic contexts with the aim to support the efforts of countries as they work toward transforming food systems to deliver on sustainable healthy diets, contributing to the achievement of the SDGs at the country level (57). Harmonized data from the Balkan region and the assessment of dietary intake will contribute to the analyses of the diet on the national or regional levels according to the FAO/WHO guiding principles as well as to EAT-Lancet recommendations (58), and propose actions for the transition to a sustainable healthy diet.

Harmonized food consumption data at the individual level in Balkan region are the basis for improving the accuracy of exposure assessments, and support nutrition surveillance, diet- and health-related studies, longitudinal monitoring of the dietary patterns and nutrient intake, and evaluation of the dietary changes in the future period, nutrition epidemiology and public health nutrition, development of dietary guidelines, and evidence-based food and nutrition policy (59). The harmonized food consumption data from WBC can contribute to the international science-policy interfaces in the design of multi-sectoral and cross-scalar policies that combine food and nutrition security, public health, environmental sustainability, social well-being, and equity (60). The European Commission High-Level Expert Group (HLEG) to strengthen the International Platform for Food Systems Science (IPFSS) within the context of the 2021 UN Food Systems Summit for food systems transformation report stressed the needs for curated and standardized data on the global level for evidence-based policy making. The European Partnership on Safe and Sustainable Food Systems for People, Planet, and Climate food systems observatory proposes harmonized monitoring efforts on the national level, with capacities and protocols to monitor and map food system drivers and outcomes across Europe, and harmonization in the Balkan region will contribute to this action (55, 61).

Capacity development in RI, especially in regionally harmonized methodology in food consumption and dietary intake assessment according to the EU Menu, will allow comparison of the eating behaviors and dietary intake of the population in WBC with other countries and regions, and further contribute to the development of the national evidence-based FBDG and dietary recommendations and monitoring of food systems transformation in the Balkan region (19). The RI and established cooperation can be further improved and employed in the development of a food-profiling model for healthy and sustainable diets, the modeling of healthy and sustainable dietary patterns, healthy and sustainable public food procurement, dietary shifts toward plant-based food products, creation of healthy digital food environments, food reformulation, and overall understanding of interconnectivity between different actors and areas of a food system (62, 63).

### Strengths and Limitations

The adaptable structure of the platform, the application of EFSA EU Menu methodology guidance to the letter and the extensive capacity development, which preceded the study, made this RI exploitable for other regions and food consumption studies. For instance, the platform can be adapted for other languages, and it uses English as the default language. It implies the use of FoodEx2 classification, which is becoming the universal food coding system worldwide. Any FCDB made according to EuroFIR™ recommendations and standards can be integrated into the platform. Moreover, the CAPNUTRA and IMR-CENM experts' team have established a certified training system for food consumption data collection studies.

Furthermore, the creation of this RI enables the strengthening of the professional scientific community, capacity development, and networking, particularly inter-country collaboration (as recommended in ICN2 FfA, No. 6) (47) between the Central and South-Eastern European countries, the EU, the Caucasus/Eurasian region, and wider research community (as emphasized in Belgrade declaration) (52). As already well-recognized capacity-building network situated in the region, CAPNUTRA with its expertise and RI plays a proactive role, validating its organizational leadership in upcoming public health nutrition and FNH-RI initiatives https://fnhri.eu/ (accessed Dec 15, 2021).

The limitation of the applied methodology lies in the fact that modern ICT solutions, such as phone/tablet apps, were not used in the process of data collection on the field, i.e., data were collected on the paper form of questionnaires and then (within 3 days) inserted into the platform. It is certain that it would be somewhat easier for the interviewers to directly insert the data as collected, but there were many concerning issues, such as burden on the respondents, insecurity regarding the internet connection availability, loss of data due to software system bugs, etc. Besides that, EFSA EU Menu methodology requires the archiving of the questionnaires for at least 5 years. Having all this in mind, the team decided to go for this “classical” way of data collection, with an ambition to work further on the development of contemporary ICT solutions, such as apps or other software tools.

## Conclusion

The CAPNUTRA network, together with the IMR-CENM team, has delivered impressive food and nutrition capacity development results in the field of public health nutrition research, RI, regionally harmonized methodology in food consumption data collection and creation of fundamental data resources for further assessment, evidence-based nutrition policymaking, and further food systems transformation in the Balkan region.

The implemented DAP platform presents successful regional cooperation in the development of harmonized methodology in compliance with EU Menu guidance. This unique platform for food consumption data collection, regional capacity development, i.e., transfer of knowledge and technical expertise, allowed for the continual and synchronized update of Regional FCDB but also for the development of other elements of contemporary RI in food and nutrition.

The DAP has essential features of RI necessary for strengthening surveillance, monitoring, evaluation, and research in the public health nutrition field. This is a rare example of a standardized and harmonized tool for dietary assessment surveys, i.e., food and nutrition data collection and analysis for evidence-based nutrition policy in Serbia and the Western Balkans. Its features make it a concurrent tool for large nutrition epidemiology studies, not only in CEE/BC but in a wider geographical context, and present one of the New Technologies for Dietary Intake Assessments. The implemented harmonized methodology in food consumption data collection in national dietary surveys in the Balkan region will contribute to the establishment of national nutrient reference values and evidence-based FBDG, which are national and regional priorities for food environment improvement, the transition to a sustainable healthy diet, and the prevention of different forms of malnutrition. According to FAO methodology, national food consumption datasets are a major resource for the further development of evidence-based FBDG.

Capacity development and harmonized methodology of food consumption collection in Balkan region will contribute to the creation of the food and nutrition policy-food system action plans for a dietary shift to a sustainable healthy diet for food systems transformation at the country or regional level based on country-specific food consumption and dietary intake data, as well as longitudinal monitoring of the dietary patterns and nutrient intake and evaluation of dietary changes in the future period and effects of the policy actions and food systems transformation monitoring.

Food consumption data from WBC, collected using improved, standardized, and harmonized methodology/tools in dietary surveys, contribute to the collection of the EFSA comprehensive European Food Consumption Database. In addition, these data can finally fill the regional gap on the FAO/WHO GIFT global food consumption database platform. Active participation in EC, EFSA research projects, and cooperation with FAO REU and other European partners and networks enhanced nutritional training, the exchange of information, knowledge transfer, and successful regional CD in food, nutrition, and public health research.

## Data Availability Statement

The raw data supporting the conclusions of this article will be made available by the authors, without undue reservation.

## Ethics Statement

The studies involving human participants were reviewed and approved by the Ethical Committee of the Institute for Medical Research, University of Belgrade on December 8th, 2017 (EO 123/2017), according to the guidelines of the Declaration of Helsinki. Written informed consent to participate in this study was provided by the participants' legal guardian/next of kin.

## Author Contributions

MGu, MN, JM, MZ, AK, and MGl contributed to the conception and design of the study. MGu and JM coordinated the workflow and writing—original draft preparation. MGu, MN, MZ, JM, and AK wrote sections of the manuscript. MR prepared graphical elements of the manuscript and edited the writing. All authors contributed to manuscript revision, reading and approved the submitted version, and have read and agreed to the published version of the manuscript.

## Funding

This work was supported by the Ministry of Education, Science, and Technological Development of the Republic of Serbia (Grant No. 451-03-9/2021-14/200015), European Food Safety Authority (EFSA) OC/EFSA/DATA/2016/02-CTO1 and OC/EFSA/DATA/2016/O3 CTO3.

## Conflict of Interest

The authors declare that the research was conducted in the absence of any commercial or financial relationships that could be construed as a potential conflict of interest.

## Publisher's Note

All claims expressed in this article are solely those of the authors and do not necessarily represent those of their affiliated organizations, or those of the publisher, the editors and the reviewers. Any product that may be evaluated in this article, or claim that may be made by its manufacturer, is not guaranteed or endorsed by the publisher.
