# Supplementary material for: Implementation of Harmonized Food Consumption Data Collection in the Balkan Region According to the EFSA EU Menu Methodology Standards
Source: Front Nutr. 2022 Jan 20;8:809328. doi: 10.3389/fnut.2021.809328 (PMC8811292; doi:10.3389/fnut.2021.809328)
Supplement: Supplementary file 1 [file Data_Sheet_1.PDF]

Diet Assess & Plan

Home | About us | Food composition database | Food intake questionnaires

Back | Copy the food into new food item

FOOD ID:	0003061
FOOD NAME (English):	Yoghurt, 2.8% mf
GENERIC ORIGINAL FOOD NAME:	Jogurt, 2.8% mlecne masti
SCIENTIFIC NAME:	
BRAND NAME:	
FOOD NAME (RS):	Jogurt, 2.8% mlecne masti
FOOD NAME (MK):	Јогурт, 2.8% млечна маст
FOOD NAME (BiH):	Jogurt, 2.8% mlecne masti
FOOD NAME (MN):	Jogurt, 2.8% mlecne masti
ORIGIN:	A
FOOD GROUP:	MILK_MILK_PRODUCT OR MILK SUBSTITUTE
FOOD SUB GROUP:	MILK, MILK PRODUCT OR MILK SUBSTITUTE
ACTIVE FOOD:	Yes
PRESENT ON SITE:	Yes
CALCULATE AS SUPPLEMENT:	No
INDUSTRIAL PRODUCT:	Yes
EXCLUDE FROM QUESTIONNAIRES:	No
ALLERGENS:	

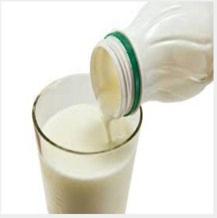

SMALL PORTION 125(g/ml) — Upload photo

MEDIUM PORTION 250(g/ml) — Upload photo

LARGE PORTION 500(g/ml) — Upload photo

EXTRA LARGE PORTION 0(g/ml) — Upload photo

*RS Admin / Serbia

EDIT DATA

NUTRITIVE VALUES | RECIPE | LANGUAL | **FOODEX 2**

Exposure hierarchy

- Foodex2 codes
  - Grains and grain-based products[A000J]
  - Vegetables and vegetable products[A00FJ]
  - Starchy roots or tubers and products thereof, sugar plants[A00
  - Legumes, nuts, oilseeds and spices[A011X]
  - Fruit and fruit products[A01BS]
  - Meat and meat products[A01QR]
  - Fish, seafood, amphibians, reptiles and invertebrates[A026T]
  - Milk and dairy products[A02LR]
  - Eggs and egg products[A031E]
  - Sugar and similar, confectionery and water-based sweet desser
  - Animal and vegetable fats and oils and primary derivatives the

BASIC CODE		
A02NG	Yoghurt, cow milk, plain	
**FACET : [F02] Part-nature**		
A0BZ2	Fermented milk (as part-nature)	
**FACET : [F07] Fat-content**		
A06ZP	2.8 % fat	
**FACET : [F10] Qualitative-info**		
A077A	Full fat	
**FACET : [F27] Source-commodities**		
A04HG	Cattle milk	
**FACET : [F28] Process**		
A0CQZ	Fermentation	

Search | ADD FoodEX2 CODE to FOOD

© 2011-2019 DAP. All rights reserved.
