# Supplementary material for: Implementation of Harmonized Food Consumption Data Collection in the Balkan Region According to the EFSA EU Menu Methodology Standards
Source: Front Nutr. 2022 Jan 20;8:809328. doi: 10.3389/fnut.2021.809328 (PMC8811292; doi:10.3389/fnut.2021.809328)
Supplement: Supplementary file 2 [file Data_Sheet_2.PDF]

Diet Assess & Plan

Time of consumption (hh.mm)	10.00
Meal type	Snack btw breakfast and lunch
Place of consumption	At home
FOOD/RECIPE code	... 0003294 **Biscuit, Jaffa / Jaffa keks**
Food/Recipe Description	Jaffa keks
Recipe (yes/no)	No
Consumed amount (g/ml)	30.00
Supplement (yes/no)	No
Brand name	
Comment	
Consumption registration item not completed	☐
Added ingredient	

SELECTED FOODEX2 FACET:

- Artificial sweeteners (e.g., aspartam, saccharine)[A046M]
- Sugars and similar[A0BY6]

Sweetening		
F08.A032K	Refined beet sugar	✖
F08.A034D	Sugar beet syrup	✖
**Fortified**		
**Qualitative info**		
**Packaging materijal**		
F19.A16RX	Polypropylene (PP)	✖

© 2011-2017 CapNUTRA. All rights reserved.
